# Rapid progression of cervical squamous cell carcinoma with delayed treatment in pregnancy

**DOI:** 10.1016/j.gore.2022.100960

**Published:** 2022-03-16

**Authors:** Luke Schmidt, Adam Crosland, Diana Pearre, Jill Tseng, Jennifer Jolley

**Affiliations:** Department of Obstetrics and Gynecology at University of California, Irvine School of Medicine in Orange California, United States

**Keywords:** Cervical cancer, Pregnancy, Disease progression, Delayed treatment, Squamous cell, Carcinoma, Surveillance

## Abstract

• Delayed treatment of cervical cancer in pregnancy can result in progression.
• Surveillance of cervical cancer in pregnancy with pelvic MRIs every 6 weeks.
• Comprehensive multidisciplinary care is essential in setting of treatment delays.

## 1 Introduction

As the peak incidence for childbearing begins to shift to the third decade of life, there is an increased chance for pregnancy to overlap with common malignant neoplasms (Perrone et al., 2019). Cancer in pregnancy introduces increasing medical, psychological, and treatment challenges that are distressing to both the pregnant patient and clinician. More specifically, cervical cancer is the leading gynecologic cancer implicating pregnancy, complicating 1.4–4.6 per 100,000 pregnancies (Amant et al., 2019, Hunter et al., 2008). The management of cervical cancer in pregnancy is influenced by multiple factors including the International Federation of Gynecology and Obstetrics (FIGO) stage and tumor size, nodal status, the histologic subtype of the tumor, and gestational age of the fetus at the time of diagnosis. Furthermore, the ability to reference well-published guidelines is limited by the difficulty in researching such a complex topic. Based on these factors, women with early stage macroscopic invasive cervical carcinoma diagnosed prior to fetal viability have historically been recommended to terminate the pregnancy for immediate definitive cancer treatment to both optimize their cancer prognosis and mitigate potential maternal morbidity and mortality (Hunter et al., 2008). Notably, disease progression associated with planned treatment delay in pregnancy is not common (Takushi et al., 2002 Nov). Additionally, there is significant consideration of the patient’s wishes regarding the continuation of pregnancy and delaying treatment in recognition of complex cultural, ethical, religious and personal dilemmas inherent to influencing this decision (Hunter et al., 2008, Gurney and Blank, 2009).

For pregnant patients with IB1 cervical cancer who postpone treatment, radical abdominal hysterectomy with bilateral pelvic lymphadenectomy following cesarean delivery remains the standard of care. Furthermore, adjuvant radiotherapy is also recommended in women with high-risk tumor characteristics (Hunter et al., 2008, Gurney and Blank, 2009). For pregnant patients with IB2 and IB3 cervical cancer who elect to continue the pregnancy, neoadjuvant chemotherapy during early to mid-second trimester is the recommended therapy (Hunter et al., 2008, Song et al., 2019).

Thus, while patients may delay treatment until after delivery or decline recommended treatment, it is still not definitively known how lengthy treatment delay will affect disease progression and the prognosis of either the mother or the infant. Additionally, clear guidelines for close surveillance of cervical cancer disease progression in pregnant patients who delay treatment is not well delineated. We present a case of a patient with FIGO stage IB1 squamous cell carcinoma (SCC) of the cervix diagnosed at 8 weeks’ gestation who declined neoadjuvant chemotherapy and was expectantly managed with serial imaging and pelvic exams. Ultimately, the patient underwent a Cesarean radical hysterectomy at 32 weeks’ gestation following rapid progression of the tumor to FIGO stage IB3 detected on surveillance imaging.

## 2 Case report

A 31-year-old Hispanic primigravida first presented to her primary care physician due to post-coital bleeding. Her gynecologic history was significant for no previous cervical cancer screening with Papanicolaou (pap) smears nor receiving the human papillomavirus (HPV) vaccination series. Atypical squamous cells of undetermined significance (ASCUS) were detected on Pap smear at time of conception. Several biopsies were obtained from the anterior and posterior cervical lesions which ultimately confirmed squamous cell carcinoma (SCC) of the cervix. Her past medical, surgical, and family histories were noncontributory. She had recently moved from Mexico to the United States and had no medical insurance. Following the aforementioned evaluation, she was referred at 8 weeks’ gestation for gynecologic oncology consultation after initial diagnosis of biopsy-proven SCC of the cervix.

**Fig. 1:**
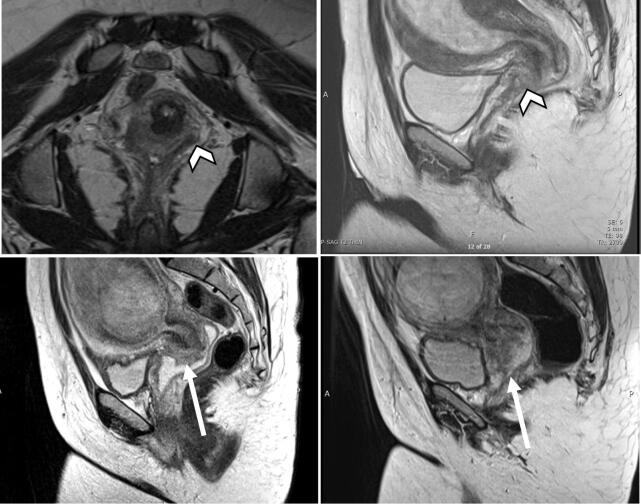
2018 FIGO stage IB1 disease progression to IB3 disease. **(a, b)** Axial oblique **(a)** and sagittal **(b)** T2-weighted image depicts (arrowhead) an exophytic cervical mass involving the anterior lip measuring 1.6 × 1.8 × 1 cm. **(b)** Sagittal T2-weighted image reveals (arrow) enlarging exophytic cervical mass measuring 1.8 × 3 × 1 cm. **(c)** Sagittal T2-weight image depicts (arrow) enlarging exophytic cervical mass of the anterior lip measuring 3.2 × 4.2 × 1.8 cm.

At her initial consultation, a speculum exam revealed an approximately 2 cm fungating cervical mass emanating from the 10 to 12o’clock position without parametrial induration or vaginal lesions. This classified the lesion between FIGO stage IB1 versus IB2 and further imaging was pursued to assess extent of disease and nodal status (Bhatla et al., 2019). Magnetic resonance imaging (MRI) of the pelvis demonstrated an exophytic cervical mass involving the anterior lip measuring 1.6 × 1.8 × 1 cm without extracervical extension or lymphadenopathy (Fig. 1a-b). Based on clinical exam and MRI the tumor was ultimately considered to be FIGO stage IB1 (Bhatla et al., 2019).

Multidisciplinary counseling with a gynecologic oncologist and maternal-fetal medicine (MFM) specialist was coordinated to counsel and review the current management and treatment recommendations. The patient was advised that pregnancy termination and immediate radical hysterectomy with lymphadenectomy, or neoadjuvant chemotherapy with radical hysterectomy at time of Cesarean delivery, were considered standard treatment recommendations. The possibility of close observation with serial MRIs was also discussed given stage IB1 disease. The patient was very reluctant to pursue treatment during pregnancy citing a recent, untimely death of her mother. Ultimately, the patient elected to continue her pregnancy without intervention. A plan was made to move forward with close surveillance with serial MRI imaging every 6 weeks and monthly pelvic examinations. Delivery was recommended via scheduled cesarean section with the potential to receive antenatal steroid for fetal lung maturity if there was a concern for disease progression while the fetus was still considered premature. The patient was also counseled that if there is evidence of tumor growth, neoadjuvant chemotherapy would also be recommended as standard treatment.

On follow up visit at 20 weeks’ gestation, pelvic examination revealed progression. This was corroborated by pelvic MRI demonstrating tumor size progression to 1.8 × 3 × 1 cm (Fig. 1c). The parametria remained uninvolved and there was no evidence of lymphadenopathy. Given the tumor size and interval growth over a short period of time in the second trimester, chemotherapy was again recommended as an interim treatment until a radical hysterectomy at 36–37 weeks’ gestation. The patient was extensively counseled regarding the risks of delaying treatment. Because the patient continued to decline chemotherapy without radiologic or clinical evidence of extracervical tumor extension, close observation with serial MRIs and examinations was continued.

Repeat pelvic and abdominal MRI at 29 weeks’ gestation demonstrated continued tumor growth, now measuring 3.2 × 4.2 × 1.8 cm (Fig. 1d) without parametrial, nodal, or abdominal involvement. With these new clinical and radiologic findings concerning for disease progression to stage IB3, a multidisciplinary meeting with gynecology oncology, MFM, radiation oncologists, and pediatricians was convened. She was counseled on her options including examination under anesthesia with possibility of radical hysterectomy, versus expectant management with chemoradiation therapy 6 weeks after delivery. Ultimately, the patient elected to proceed with a Cesarean delivery at 32 weeks’ gestation and possible radical hysterectomy based on the tumor findings at the time of examination under anesthesia. She was then admitted to labor and delivery for administration of antepartum steroids for fetal lung maturity and neonatology consultation.

**Fig. 2:**
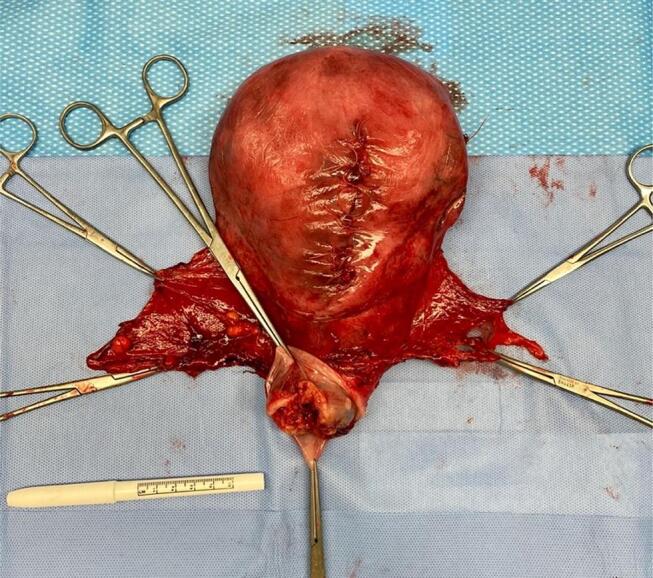
Gross anatomical depiction of hysterectomy specimen with exophytic cervical tumor involving the anterior lip (arrow).

During the examination under anesthesia, a 4 cm cervical tumor was noted to be exophytic with a 1 cm border of normal-appearing cervix proximal to the lesion. Given the rapid growth of the tumor, there was a concern that postponing chemo-radiation until 6 weeks after cesarean delivery would significantly delay the initiation of her definitive care. After a multidisciplinary conference between gynecology oncology, radiation oncology, MFM, and radiology was held to discuss this aforementioned concern, the decision was made to proceed with exploratory laparotomy, Cesarean-radical hysterectomy, bilateral salpingectomy, bilateral pelvic lymphadenectomy, ovarian transposition, and cystoscopy. The patient was counseled that she would likely need post-operative radiation therapy. Following the uncomplicated delivery of a healthy male infant, intraoperative pelvic survey was unremarkable and there was no evidence of parametrial or vaginal invasion nor any extra-cervical spread of disease. Intraoperative frozen pathology of bilateral pelvic lymph nodes were negative. The hysterectomy specimen (Fig. 2) revealed a grade 3 poorly differentiated invasive squamous cell carcinoma, HPV-associated. The specimen measured 5.5 cm in the greatest dimension. The depth of invasion was at least 20 mm, lymphovascular invasion was present, and all resection margins were free of carcinoma.

On postoperative day 3, the patient noted left lower extremity pain that radiated from her groin down to her leg and was diagnosed with a non-occlusive deep vein thrombosis of the left common femoral vein despite initiation of prophylactic enoxaparin sodium on postoperative day 1. She was subsequently started on therapeutic enoxaparin sodium which were later confirmed to be therapeutic prior to discharge on postoperative day 6. The patient did meet Sedlis criteria and went on to receive radiation therapy 6 weeks after her radical hysterectomy and bilateral pelvic lymphadenectomy (Sedlis et al., 1999 May). A 2 cm nodule located on the anterior vaginal wall, just distal to the apex, was found on physical exam and corroborated with imaging 3 months following her radical hysterectomy—confirming vaginal recurrence. She then underwent exploratory laparotomy, left ureterolysis, resection of pelvic sidewall tumor, cystoscopy, left double J-ureteral stent placement, omental J-flap, bilateral fasciocutaneous flaps with incisional closure, and intraoperative radiotherapy to the left pelvic sidewall. Most recently the patient has completed 3 cycles of carboplatin and paclitaxel, and continues receiving pembrolizumab therapy.

## 3 Discussion

Reports of progression of stage IB cervical cancer in pregnancy are rare (Takushi et al., 2002 Nov). It is not definitively known how a planned delay in pregnant patients with stage IB disease will affect the prognosis and survival of the mother or infant. There is no standard definition on what constitutes an acceptable duration of treatment delay in these patients due to the absence of substantial data. Studies have indicated no disease progression with treatment delays up to 40 weeks in patients with stage I cervical cancer (Sorosky et al., 1995 Nov). In a prospective study by Sorosky et al including 8 pregnant patients with stage I squamous cell carcinoma, there was no significant disease progression noted in patient-elected treatment delays between 3 and 40 weeks (range 21–282 days; median 112 days) (Sorosky et al., 1995 Nov). Additionally, Duggan et al reported a mean diagnosis-to-treatment interval of 144 days (range 53–212) in 8 patients with stage IA or IB cervical cancer who elected to delay treatment (Duggan et al., 1993). After a median follow-up of 33 months, none of the patients had disease recurrence. Takushi et al reported no disease progression noted in patient-elected treatment delays between 6 and 15 weeks in 4 patients with stage IA2 to IB2 cervical cancer (Takushi et al., 2002 Nov). Finally, an additional study also reported delays of up to 32 weeks in patients with early-stage lesions without an obvious compromise in overall survival (Sood et al., 1996). Conversely, Dudan et al reported two pregnant patients diagnosed with IB cervical cancer who deliberately delayed therapy and were subsequently noted to have disease progression to IIA and IIIB disease postpartum (Dudan et al., 1973). Additionally, Nisker and Shubat reported the death of one patient who was diagnosed with stage IB cervical cancer and elected to delay her treatment for 24 weeks (Nisker and Shubat, 1983). Collectively, all of the aforementioned studies overall support delay in treatment to allow for fetal maturity in patients with early Stage I cervical cancer in pregnancy; however, there is still an insufficient number of patients in literature to support a definitive conclusion. This case highlights the importance of the length of treatment delay when patients are considering planned delay in treatment of cervical cancer in pregnancy given disease progression in pregnancy is rare but can occur with significantly delayed treatment.

While there is growing supportive evidence that neoadjuvant chemotherapy should be counseled to patients who want to continue their pregnancy in setting of locally advanced disease diagnosed in the early to mid second trimester to prevent disease progression, a steadfast patient population desiring lengthy treatment delays is still anticipated due to the complex cultural, ethical, and personal dilemmas embedded in an individual’s decision-making process, especially in the context of considering maternal versus fetal outcome (Song et al., 2019, Tewari et al., 1998, Marana et al., 2001, Halaska et al., 2019). In a recent cohort of 132 pregnant patients with cervical cancer, 26.5% of the patients decided to delay treatment upon diagnosis, predominantly including patients with IB1 and IB2 tumors (78.3%) (Halaska et al., 2019). For treatment during pregnancy, only 17.4% of the patients underwent surgery and 16.7% received neoadjuvant chemotherapy (Halaska et al., 2019). The ability of providers to effectively deliver health care services that meet the social and cultural needs of patients delaying treatment of cervical cancer during pregnancy is of upmost importance in order to construct a culturally competent health care system that reduces negative health consequences.

The prevalence of patients delaying treatment of cervical cancer in pregnancy despite growing support for neoadjuvant chemotherapy draws attention to the importance of standardizing surveillance methods when treatment is delayed, especially due to the possibility of disease progression as noted in this case. There is currently no standardized surveillance method for pregnant patients with cancer. Duggan et al advocated the use of serial pelvic MRIs in the assessment of tumor size and spread (Duggan et al., 1993). A case report by Gurney and Blank documented close surveillance of a pregnant patient delaying cervical cancer treatment which included serial colposcopy exams in addition to serial pelvic MRIs (Gurney and Blank, 2009). In this case report, largely due to a gross visible lesion, less invasive surveillance with monthly pelvic exams and pelvic MRIs every 6 weeks successfully identified disease progression. Additional studies evaluating the most efficacious surveillance modalities and frequencies would be beneficial to inform a standardized surveillance method. This would enable medical providers to better counsel pregnant patients with cancer who are interested in delaying treatment, more accurately inform the patient and medical team throughout the course of treatment delay and prevent missed treatment options preferential to improving the prognosis of mother and infant. Overall, a standardized surveillance method of cervical cancer in pregnancy would provide a strategy for improving culture competence in the health care services provided to patients delaying treatment of cervical cancer in pregnancy and improve their quality of care.

The incidence of cervical cancer during pregnancy is overall low and therefore there is a paucity of data regarding disease progression in pregnancy, which complicates our ability to fully discuss prognosis for those electing treatment delay in pregnant patients with stage IB SCC of the cervix. We report a case that demonstrates disease progression from stage IB1 to IB3 SCC of the cervix in a pregnant patient who delayed treatment for 24 weeks, highlighting the importance of strict compliance with close surveillance to prevent the limitation of potential treatment options associated with better prognosis for mother and infant. This case also offers providers useful insight on navigating uniquely dynamic treatment planning and complex counselling in the setting of lengthy treatment delays, including comprehensive multidisciplinary counseling, non-invasive close surveillance methods, and the unmeasurable effects of cultural competency.

## Author contributions

All authors contributed to the conceptualization of the presented case report. L.S. wrote the initial draft of the case report with input from all authors. L.S, A.C. and D.P. made substantial contribution to the design of the case report. All authors drafted or critically revised the case report for important content, approved the version to be published, and agree to integrity or accuracy of any part of the work. J.T. and J.J. provided oversight and leadership responsibility for the case report execution. A.C. contributed significantly to resources for case report execution.

## Declaration of Competing Interest

The authors declare that they have no known competing financial interests or personal relationships that could have appeared to influence the work reported in this paper.

## References

[b0005] Perrone A.M., Bovicelli A., D'Andrilli G., Borghese G., Giordano A., De Iaco P. (2019). Cervical cancer in pregnancy: analysis of the literature and innovative approaches. J. Cell. Physiol..

[b0010] Amant F., Berveiller P., Boere I.A., Cardonick E., Fruscio R., Fumagalli M., Halaska M.J., Hasenburg A., Johansson A.L.V., Lambertini M., Lok C.A.R., Maggen C., Morice P., Peccatori F., Poortmans P., Van Calsteren K., Vandenbroucke T., van Gerwen M., van den Heuvel-Eibrink M., Zagouri F., Zapardiel I. (2019). Gynecologic cancers in pregnancy: guidelines based on a third international consensus meeting. Ann. Oncol..

[b0015] Hunter M.I., Tewari K., Monk B.J. (2008). Cervical neoplasia in pregnancy. Part 2: current treatment of invasive disease. Am J Obstet Gynecol..

[b0020] Takushi M., Moromizato H., Sakumoto K., Kanazawa K. (2002). Management of invasive carcinoma of the uterine cervix associated with pregnancy: outcome of intentional delay in treatment. Gynecol Oncol..

[b0025] Gurney E.P., Blank S.V. (2009). Postpartum radical trachelectomy for IB1 squamous cell carcinoma of the cervix diagnosed in pregnancy. Am J Obstet Gynecol..

[b0030] Song Y., Liu Y., Lin M., Sheng B., Zhu X. (2019). Efficacy of neoadjuvant platinum-based chemotherapy during the second and third trimester of pregnancy in women with cervical cancer: an updated systematic review and meta-analysis. Drug Des. Develop. Ther..

[b0035] Bhatla N., Berek J., Cuello M. (2019). New revised FIGO staging of cervical cancer (2018). Int. J. Gynecol. Obstet..

[b0040] Sedlis A., Bundy B.N., Rotman M.Z., Lentz S.S., Muderspach L.I., Zaino R.J. (1999). A randomized trial of pelvic radiation therapy versus no further therapy in selected patients with stage IB carcinoma of the cervix after radical hysterectomy and pelvic lymphadenectomy: a Gynecologic Oncology Group Study. Gynecol. Oncol..

[b0045] Sorosky J.I., Squatrito R., Ndubisi B.U., Anderson B., Podczaski E.S., Mayr N., Buller R.E. (1995). Stage I squamous cell cervical carcinoma in pregnancy: planned delay in therapy awaiting fetal maturity. Gynecol. Oncol..

[b0050] Duggan B., Muderspach L.I., Roman L.D., Curtin J.P., d'Ablaing G., Morrow P.C. (1993). Cervical cancer in pregnancy: reporting on planned delay in therapy. Obst. Gynecol..

[b0055] Sood A.K., Sorosky J.I., Krogman S., Anderson B., Benda J.o., Buller R.E. (1996). Surgical management of cervical cancer complicating pregnancy: a case-control study. Gynecol. Oncol..

[b0060] Dudan R.C., Yon J.L., Ford J.H., Averette H.E. (1973). Carcinoma of the cervix and pregnancy. Gynecol. Oncol..

[b0065] Nisker J.A., Shubat M. (1983). Stage IB cervical carcinoma and pregnancy: report of 49 cases. Am. J. Obstet. Gynecol..

[b0070] Tewari K., Cappuccini F., Gambino A., Kohler M.F., Pecorelli S., DiSaia P.J. (1998). Neoadjuvant chemotherapy in the treatment of locally advanced cervical carcinoma in pregnancy: a report of two cases and review of issues specific to the management of cervical carcinoma in pregnancy including planned delay of therapy. Cancer.

[b0075] Marana H.R.C., Moreira de Andrade J., do Carmo da Silva Mathes Â., Duarte G., Pereira da Cunha S., Bighetti S. (2001). Chemotherapy in the treatment of locally advanced cervical cancer and pregnancy. Gynecol. Oncol..

[b0080] Halaska M.J., Uzan C., Han S.N., Fruscio R., Dahl Steffensen K., Van Calster B., Stankusova H., Delle Marchette M., Mephon A., Rouzier R., Witteveen P.O., Vergani P., Van Calsteren K., Rob L., Amant F. (2019). Characteristics of patients with cervical cancer during pregnancy: a multicenter matched cohort study. An initiative from the International Network on Cancer, Infertility and Pregnancy. Int. J. Gynecol. Cancer.

